# Alternations of gut microbiota composition in neonates conceived by assisted reproductive technology and its relation to infant growth

**DOI:** 10.1080/19490976.2020.1794466

**Published:** 2020-08-04

**Authors:** Qun Lu, Yuan Lin, Ting Chen, Hong Lv, Feiyang Diao, Cong Liu, Meijuan Peng, Xiufeng Ling, Hong Li, Yun Wang, Yongyue Wei, Jiangbo Du, Guangfu Jin, Yankai Xia, Hongxia Ma, Xingyin Liu, Hongbing Shen, Zhibin Hu

**Affiliations:** a State Key Laboratory of Reproductive Medicine, Nanjing Medical University, Nanjing, China; b Department of Epidemiology and Biostatistics, School of Public Health, Nanjing Medical University, Nanjing, China; c Department of Maternal, Child and Adolescent Health, School of Public Health, Nanjing Medical University, Nanjing, China; d Scientific Education Section, The Affiliated Nanjing Maternity and Child Health Hospital of Nanjing Medical University, Nanjing, China; e Department of Reproduction, The First Affiliated Hospital of Nanjing Medical University, Nanjing, China; f Department of Reproduction, The Affiliated Nanjing Maternity and Child Health Hospital of Nanjing Medical University, Nanjing, China; g Reproductive Genetic Center, Suzhou Affiliated Hospital of Nanjing Medical University, Suzhou Municipal Hospital, Suzhou, China; h Department of Obstetrics, Suzhou Affiliated Hospital of Nanjing Medical University, Suzhou Municipal Hospital, Suzhou, China; i China International Cooperation Center for Environment and Human Health, Nanjing Medical University, Nanjing, China

**Keywords:** Assisted reproductive technology (ART), Birth Cohort, meconium microbiome, bacteroidetes, weight gain

## Abstract

The gut microbiome in newborns may be strongly influenced by their intrinsic host microenvironmental factors (e.g., the gestational age) and has been linked to their short-term growth and potentially future health. It is yet unclear whether early microbiota composition is significantly different in newborns conceived by assisted reproductive technology (ART) when compared with those who were conceived spontaneously. Additionally, little is known about the effect of gut microbiota composition on weight gain in early infancy. We aimed to characterize the features and the determinants of the gut microbiome in ART newborns and to assess the impact of early microbiota composition on their weight gain in early infancy in mother-infant dyads enrolled in the China National Birth Cohort (CNBC). Among 118 neonates born by ART and 91 neonates born following spontaneous conception, we observed significantly reduced gut microbiota α-diversity and declined Bacteroidetes relative abundance in ART neonates. The microbiota composition of ART neonates was largely driven by specific ART treatments, hinting the importance of fetus intrinsic host microenvironment on the early microbial colonization. Following up these neonates for six months after their births, we observed the effects of gut microbiome composition on infant rapid weight gaining. Collectively, we identified features and determinants of the gut microbiota composition in ART neonates, and provided evidence for the importance of microbiota composition in neonatal growth.

## Introduction

The acquisition and development of infant microbiome are key to establishing a healthy host-microbiome symbiosis.^1^ Despite the debates on whether the first microbiota colonization occurs in utero or perinatally,^2-4^ mounting evidence has suggested that infant’s intrinsic host microenvironmental factors (e.g., the gestational age) combining with exogenous factors (e.g., the mode of delivery) drive early life formation and maturation of microbial, and intrinsic factors appear to exert more crucial roles.^5-7^ Thus, prenatal stage is pivotal, in which the development of biological system may determine the selection and formation of infant’s microbiome and influence immune system development, metabolic function and potentially future health.^8^

Considerable efforts have focused on cataloging infant gut microbiome and investigating its relation to childhood development and health.^9^ However, to the best of our knowledge, no published study has specifically investigated the gut microbiome in infants conceived by the assisted reproductive technology (ART). ART, a technique that uses medical techniques and methods manually manipulate gametes, zygotes, and embryos to achieve the purpose of conception, has become a standard and common practice in reproductive medicine clinics for infertile couples worldwide. The total number of newborns in the world through ART has exceeded five million.^10^ ART treatment includes multiple procedures, e.g., maternal medication usage to induce ovulation or to maintain the pregnancy in the early stages, procedures handling both the egg and sperm outside body, as well as freezing and thawing of embryos. Previous studies showed that these procedures have potential impact on maternal microenvironment and embryos,^11^ hence biologically plausible to cause an alternation of gut microbiome in offspring. For example, progesterone, the main component of luteal phase support drug used in ART treatment, has been reported alter gut microbial composition in mice during gestation in recent publication.^12^ Additionally, ART pregnancies are at a higher risk for preterm delivery,^13^ which is an intrinsic factor likely associated with the degree of maturation and stability of newborn’s gut microbiome.^6^ The bacteria found in neonates’ first meconium reflects the earliest colonization and formation of microbiota, and then neonates’ gut microbiota is gradually shaped by dietary, nutrition, and medical factors in the following days.^7,8,14^ It is yet not clear whether early microbiota composition is significantly different in infants conceived by ART when compared with those who were conceived spontaneously. Additionally, the impact of neonatal microbial composition on their growth and development is still mostly unknown.

Therefore, to expand upon the understanding of the change of gut microbiota structure between ART and spontaneous groups, we recruited mothers who conceived spontaneously and those who conceived after ART, and sampled the first meconium and the fecal samples at the second, third and fourth day of age afterward from their newborns. The 16S rRNA gene sequencing was used to evaluate the microbial population between ART- and spontaneous-neonates. To evaluate the effect of different procedures related to ART on microbial composition of newborns, microbiota community changes across different procedures in ART-neonates were further investigated. Furthermore, a correlation analysis between neonatal growth and individual bacteria was also conducted to explore the associations among these differential species and neonatal growth.

## Results

### Study population

To characterize the early gut microbiome in neonates born after ART, we profiled the microbiome in the first meconium and in the fecal samples collected at the second, third, and fourth day after birth from 209 singleton neonates (118 conceived by ART, and 91 conceived spontaneously) in the China National Birth Cohort (CNBC). All mothers in this cohort were recruited prospectively before receiving ART procedure (ART conception) or at first trimester of gestation (spontaneous conception), and their offspring were followed up during the course of infancy. In the present study, 57% (*N* = 67) neonates in the ART conception group and 46% (*N* = 42) in the spontaneous conception group were males. Among the ART neonates, 75 were conceived through invitro fertilization (IVF) and 43 were through intracytoplasmic sperm injection (ICSI), and the majority (N = 84) were conceived via frozen-embryo transfer. Compared with the neonates conceived spontaneously, the ART offspring were more likely born via cesarean section (85% for ART conception versus 44% for spontaneous conception, χ2 test, *P* = 5.06 × 10^−10^), and these infants had a shorter gestational age (mean: 38.3 for ART conception versus 39.5 for spontaneous conception, student’s t test, *P* = 7.30 × 10^−10^). However, the neonatal birth weight and maternal age were comparable between ART and spontaneous conception groups (Table 1).

**Table 1.** Demographic and clinical characteristics of the participants.

Characteristics	ART (*n* = 118)	Spontaneous (*n* = 91)	*P* value
**Maternal**			
Age (years)	30.47 ± 3.70	29.76 ± 4.34	2.14 × 10^−01^
Pre-pregnancy BMI (kg/m^2^)	21.70 ± 2.91	21.74 ± 4.14	9.47 × 10^−01^
Gravidity			3.69 × 10^−01^
0	54(45.76)	36(39.56)	
> 0	64(54.24)	55(60.44)	
**Infant**			
Mode of delivery			5.06 × 10^−10^
Cesarean section	100(84.75)	40(43.96)	
Vaginal	18(15.25)	51(56.04)	
Gender			1.27 × 10^−01^
Male	67(56.78)	42(46.15)	
Female	51(43.22)	49(53.85)	
Gestational age (weeks)	38.28 ± 1.61	39.49 ± 0.90	7.30 × 10^−10^
Birth weight (g)	3225.85 ± 492.35	3255.28 ± 485.11	6.66 × 10^−01^
Feeding mode			9.53 × 10^−02^
Exclusively breast-fed (%)	51(43.22)	51(56.04)	
Exclusively formula-fed (%)	4(3.39)	5(5.49)	
Mixed fed (formula + breast-feeding) (%)	63(53.39)	35(38.46)	
**ART processing**			
Regimen for ovulation induction			
Antagonist regimen	26(22.03)		
Microstimulation	10(8.47)		
Agonist long regimen	29(24.58)		
Short-acting long regimen	53(44.92)		
HCG_E2 (ng/mL)	3.96 ± 2.38		
HCG_P (ng/mL)	0.94 ± 0.90		
HCG_Endometrial thickness (mm)	10.35 ± 2.53		
Corpus luteum support drugs through the vagina			
no	27(22.88)		
yes	91(77.12)		
Oral or injection of corpus luteum support drugs			
no	10(8.47)		
yes	108(91.53)		
Insemination method			
IVF	75(63.56)		
ICSI	43(36.44)		
Transplant embryo type			
Fresh	34(28.81)		
Frozen	84(71.19)		

*N* = 209 dyads from the CNBC cohort. Data are presented as mean ± SD or counts and percentages, respectively. Continuous variables were tested by Student’s *t* test. Nominal variables were tested either with χ2 test or Fisher’s exact test.

BMI, body mass index; HCG_E2, Estradiol level at HCG day; HCG_P, Progesterone level at HCG day; HCG_Endometrial thickness, Endometrial thickness at HCG day.

### The first meconium microbiome from ART neonates displayed reduced α-diversity

**Figure 1.**
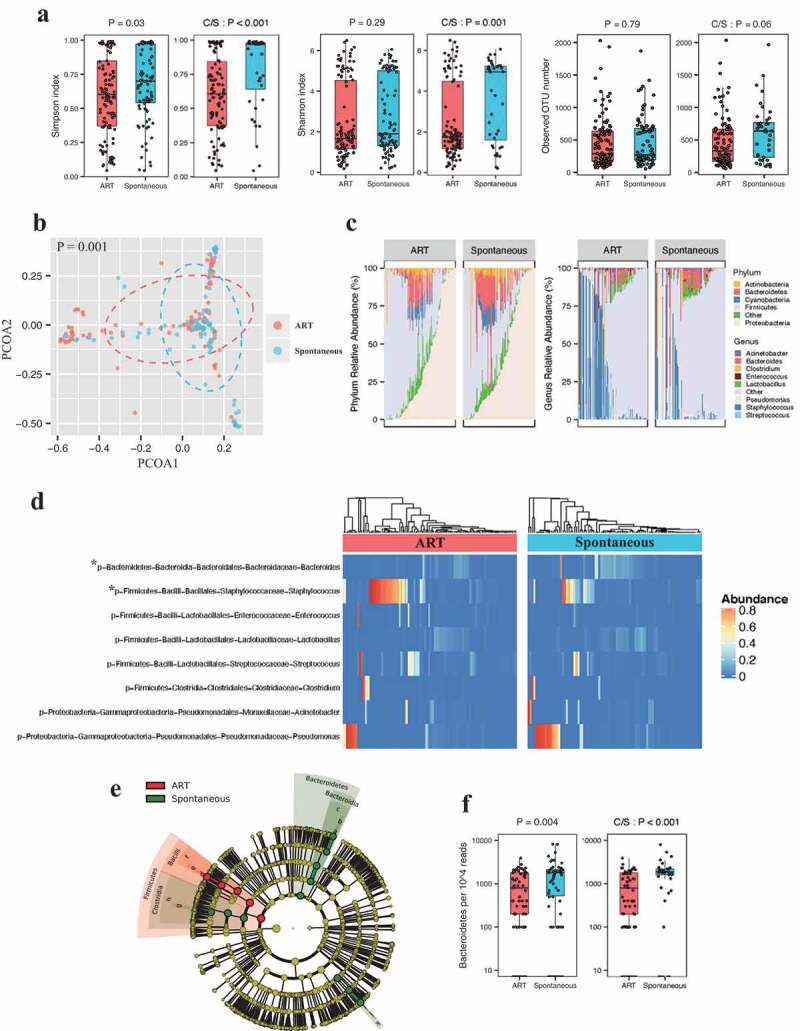
The first meconium microbiome exhibit discrete composition between ART and spontaneous conception groups.

The 209 first meconium samples from newborns were subjected to 16S rRNA gene amplicon sequencing (average 42,900 sequence reads per sample) and clustered into operational taxonomic units (OTUs) at 97% 16S rRNA gene sequence similarity. We observed a declined α-diversity (Simpson index) in the first meconium from ART neonates, compared with that in the samples collected from spontaneous conception group (mean ± SD of Simpson index of overall microbiota: 0.59 ± 0.29 for ART versus 0.68 ± 0.28 for spontaneous, one-way ANOVA, *P* = .03) (Figure 1a), indicating a less complex microbiota community in the ART neonates. Shannon index and observed OTU numbers were not significantly different in the two groups (Figure 1a). Given that mode of delivery have been reported impact infant gut microbiome composition, we specifically examined microbiota α-diversity in the neonates born after cesarean section and still observed lower Simpson index in the ART neonates (0.60 ± 0.29 for ART conception versus 0.78 ± 0.28 for spontaneous conception, one-way ANOVA, *P* < .001) (Figure 1a).

(a) Comparison of α-diversity (Simpson, Shannon index and Observed OTU numbers) between ART versus spontaneous conception groups (and between ART cesarean section and spontaneous cesarean section groups; C/S, cesarean section) using the one-way ANOVA. Boxes indicate interquartile range, lines indicate medians, and whiskers represent range. (b) Comparison of β diversity between ART versus spontaneous conception groups using PERMANOVA based on Bray-Curtis distances. (c) Relative abundance of core bacterial phyla and genera (> 1% mean relative abundance) in the meconium samples from ART and spontaneous conception groups. (d) Heatmap of the core bacterial genera for ART and spontaneous conception groups with corresponding Hierarchical clustering dendrogram. * genera relative abundance is significantly different (*P* < .05 after Bonferroni Correction) between neonates conceived by ART and following spontaneous conception. (e). Linear discriminant analysis of taxa enrichment between ART versus spontaneous conception groups. Criteria: Alpha value for the factorial Kruskal-Wallis test among classes < 0.05; Alpha value for the pairwise Wilcoxon test between subclasses < 0.05; LDA score for discriminative features > 4. (f). Relative abundance of Bacteroidetes (normalized to 10,000 read counts) between ART versus spontaneous conception groups (and between ART caesarean section and spontaneous caesarean section groups).

### ART neonates exhibited discrete composition in gut microbiota as compared to neonates conceived spontaneously

We conducted principal coordinate analysis (PCoA) and observed that the meconium microbiota in ART offspring appeared to cluster separately from that in neonates conceived spontaneously (PERMANOVA on Bray-Curtis dissimilarity, *P* = .001) (Figure 1b). We then estimated and visualized the interactions among the selected genera in the first-pass meconium from ART and spontaneous conception groups to assess their gut microbiota ecology. The two groups displayed similar networks, both showing abundant correlations among the genera (Supplementary Figure 1a and b). The quantification of the connections in the two microbial networks demonstrated that they shared the majority of the edges (connections), whereas the ART conception group showed less diverse closeness centrality (mean of the absolute correlation coefficients of one genera with all the others) (Supplementary Figure 1c).

In the neonates born following spontaneous conception, the most abundant phylum in their meconium samples was the Proteobacteria (mean ± SD relative abundance: 41.4% ± 36.3%) and then the Firmicutes (37.3% ± 32.8%), while the majority of OTUs belong to the Firmicutes in ART neonates (48.7% ± 35.8%), and then followed by the Proteobacteria (36.3% ± 36.0%). The third most dominant phylum was the Bacteroidetes in both populations, whereas it showed only 50% amount of mean relative abundance in the ART conception versus the spontaneous conception group (5.5% ± 8.4% for ART conception versus 10.5% ± 15.7% for spontaneous conception, MaAsLin, *P*_fdr_ = 2.49 × 10^−4^). The relative abundance of the Actinobacteria and the Cyanobacteria phyla were 3%-4%, and the other phyla comprised less than 1% of the 16S rRNA gene sequence in both groups (Figure 1c, Supplementary Table 1). At the genus level, in the spontaneous conception group, the most abundant taxa was *Pseudomonas* (phylum Proteobacteria). In contrast, *Staphylococcus* (phylum Firmicutes) was most abundant in ART neonates. We observed over 2-fold increase in the relative abundance of *Staphylococcus* but more than 50% reduce of *Bacteroides* (phylum Bacteroidetes) in ART neonates meconium, compared with spontaneous conception (*Staphylococcus*: 19.4% ± 33.0% for ART conception versus 7.9% ± 18.7% for spontaneous conception, MaAsLin, *P*_fdr_ = 1.34 × 10^−1^; *Bacteroides*: 1.7% ± 3.5% for ART conception versus 4.5% ± 10.2% for spontaneous conception, MaAsLin, *P*_fdr_ = 8.19 × 10^−4^; Figure 1c,d, Supplementary Table 1).

### The relative abundance of gut Bacteroidetes declined by half in the ART neonates

LEfSe analysis showed that first-pass meconium of infants born following spontaneous conception was enriched of Bacteroides (phylum Bacteroidetes). At phylum level, Bacteroidetes in meconium exhibited the most remarkable difference in abundance between two populations- the mean read counts of Bacteroidetes in the meconium samples from the ART neonates was lower by 47.6%, and the difference was not affected by cesarean section (Figure 1e,f). The Firmicutes/Bacteroidetes (F/B) ratio is considered an important index in previous studies,^15-17^ thus we calculated the ratio in 65 ART and 51 spontaneous samples (relative abundance of Firmicutes and Bacteroidetes both > 0) and observed a significantly greater F/B ratio in ART newborns (median log F/B ratio: 0.39 for ART conception versus 0.22 for spontaneous conception, one-way ANOVA, *P* < .001) (Supplementary Figure 1d).

### Gut microbiota across the first four days after birth exhibited different variability pattern in ART neonates

We calculated the intraindividual β diversity to evaluate the dissimilarity of microbiomes on first-pass meconium with later fecal samples. The infant gut microbiomes showed a high variability between the first meconium with the following three days. Of note, we observed a lower β diversity between the first meconium and the stool samples of following days in ART-neonates, compared with that in spontaneous-neonates (the median β diversity between the first meconium and the stool samples of 2^nd^, 3^rd^, and 4^th^ day in ART neonates: 0.757, 0.923, and 0.666, respectively; in spontaneous conceived neonates: 0.965, 0.968, and 0.949, respectively. Fit linear mixed-effects models, *P* = .006 for the mode of conception. Supplementary Figure 1e), hinting the first-pass meconium microbiomes in ART-neonates were more stable and changed slowly during the first few days after birth.

### Specific ART treatments impact relative abundance of gut Bacteroidetes in offspring

Among ART neonates, no association was observed of overall α-diversity or the relative abundance of Bacteroidetes with multiple conventional factors including maternal age, gestational age, mode of delivery, and infant gender, with the exception of gravidity showing a marginal association with the relative abundance of Bacteroidetes (Ever pregnant versus never pregnant: β_adj_ = −0.05, 95% CI = −0.11, −0.002, *P* = 4.11 × 10^−2^) (Supplementary Tables 2 and 3) with Fitting linear models and Tobit regression models, respectively. We specifically investigated whether ART treatments (e.g., regimen of ovulation induction, medication usage, medical examinations, insemination methodology, and type of embryo) have impact on microbiota composition in offspring. After the adjustment for gestational age, mode of delivery and infant gender, we found that the usage of vaginal suppositories for luteal phase support (VLPS) exhibited a strong and positive association with the relative abundance of Bacteroidetes (β_adj_ = 0.11, 95% CI = 0.04,0.17, *P* = 1.79 × 10^−3^; Table 2), compared with nonvaginal corpus luteum support drugs (NVLPS)(oral administration, subcutaneous or intramuscular injection). In contrast, antagonist usage during ART process and frozen embryo transfer significantly reduced Bacteroidetes abundance in ART-neonates (antagonist regimen versus agonist long regimen: β_adj_ = −0.09, 95% CI = −0.17, −0.02, *P* = 1.90 × 10^−2^, frozen versus fresh embryo: β_adj_ = −0.06, 95% CI = −0.12, −0.01, *P* = 2.89 × 10^−2^; Table 2). In addition, high estradiol levels on the HCG day showed an inverse association with Bacteroidetes abundance after adjustment (β_adj_ = −0.01, 95% CI = −0.02, −0.002, *P* = 2.49 × 10^−2^; Table 2). We did not observe significant associations between α-diversity and embryo type or other specific treatment.

**Table 2.** The association of specific ART treatments with the microbiota composition in the ART-conceived neonates.

ART treatments	Diversity (Simpson index)^a^		Bacteroidetes abundance^a^	
	β (95% CI)	*P* value	β (95% CI)	*P* value
Polycystic ovary syndrome				
No (*n* = 104)	Ref.		Ref.	
Yes (*n* = 14)	0.09 (−0.08,0.26)	2.93 × 10^−01^	−0.04 (−0.12,0.05)	4.12 × 10^−01^
Regimen for ovulation induction				
Agonist long regimen (*n* = 29)	Ref.		Ref.	
Short-acting long regimen (*n* = 53)	−0.004 (−0.14,0.13)	9.48 × 10^−01^	−0.04 (−0.10,0.02)	2.35 × 10^−01^
Antagonist regimen (*n* = 26)	−0.01 (−0.17,-0.14)	8.61 × 10^−01^	−0.09 (−0.17,-0.02)	1.90 × 10^−02^
Microstimulation (*n* = 10)	0.06 (−0.15,0.28)	5.69 × 10^−01^	−0.03 (−0.13,0.07)	5.73 × 10^−01^
Estradiol levels on the hCG day (ng/mL) (*n* = 117)	0.01 (−0.01,0.03)	3.33 × 10^−01^	−0.01 (−0.02,-0.002)	2.49 × 10^−02^
Progesterone levels on the hCG day (ng/mL) (*n* = 118)	0.002 (−0.07,0.07)	9.49 × 10^−01^	−0.02 (−0.05,0.02)	3.59 × 10^−01^
Endometrial thickness on the hCG day (mm) (*n* = 118)	−0.01 (−0.03,0.01)	4.59 × 10^−01^	0.002 (−0.01,0.01)	6.86 × 10^−01^
Insemination method				
ICSI (*n* = 43)	Ref.		Ref.	
IVF (*n* = 75)	−0.09 (−0.20,0.02)	9.45 × 10^−02^	−0.04 (−0.10,0.01)	1.08 × 10^−01^
Transplant embryo type				
Fresh (*n* = 34)	Ref.		Ref.	
Frozen (*n* = 84)	0.07 (−0.05,0.18)	2.88 × 10^−01^	−0.06 (−0.12,-0.01)	2.89 × 10^−02^
The usage of vaginal suppositories for luteal phase support^b^				
no (*n* = 27)	Ref.		Ref.	
yes (*n* = 91)	0.09 (−0.04,0.22)	1.63 × 10^−01^	0.11 (0.04,0.17)	1.79 × 10^−03^
Oral or injection of corpus luteum support drugs^b^				
no (*n* = 10)	Ref.		Ref.	
yes (*n* = 108)	0.14 (−0.07,0.36)	1.94 × 10^−01^	0.04 (−0.06,0.15)	4.11 × 10^−01^

^a^Adjusted for gestational age, mode of delivery, and infant gender.

^b^Adjusted for gestational age, mode of delivery, infant gender, and transplant embryo type.

**Figure 2.**
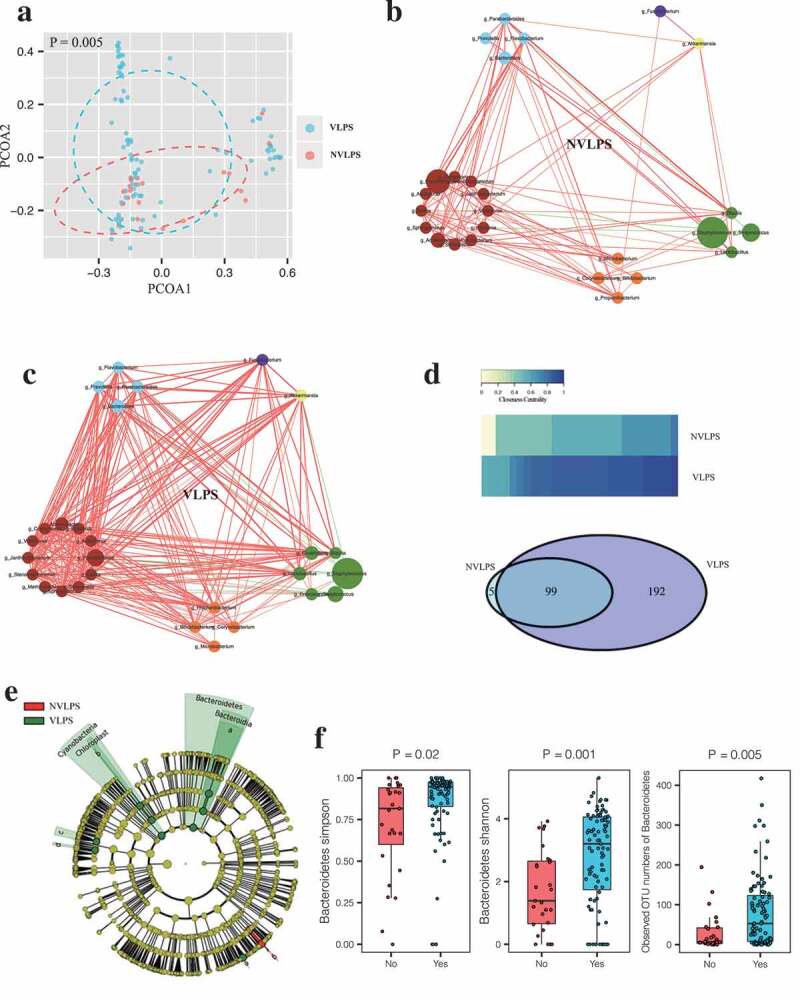
Associations between vaginal luteal support and gut Bacteroidetes abundance in the neonates conceived by ART.

The PCoA plots based on OTUs showed separated clusters for infants born to mothers who used vaginal suppositories versus those born to mothers who used nonvaginal corpus luteum support drugs (Figure 2a), and the microbial community of the vaginal drug usage group featured a more complex network (Figure 2b,c). Notably, although the majority edges (connections) were overlapped between the two groups and the vaginal drug group covered over 95% of the connections (99 out of 104) displayed in the nonvaginal drug group, 192 connections were specific to the vaginal drug group (Figure 2d). Moreover, we observed more complex closeness centrality among the genera in the vaginal drug group (Figure 2d). The LDA of taxa enrichment showed that the usage of vaginal suppositories for luteal phase support was associated with differential relative abundance for a few individual taxa in ART-offspring including Bacteroidetes and Cyanobacteria (Figure 2e). Neonates born to mothers who used vaginal suppositories exhibited increased Simpson, Shannon index and observed OTU numbers of Bacteroidetes compared with those whom born to mothers used nonvaginal corpus luteum support drugs (figure 2f).

(a) Comparison of β diversity between vaginal (VLPS) versus nonvaginal (NVLPS) luteal support drug groups using PERMANOVA based on Bray-Curtis distances. (b) Cooccurrence network analysis in NVLPS group. Each node represents a genus, nodes of the same color belong to the same phylum, the significantly correlated genera (FDR < 0.05) were visualized. The node size represents the relative abundance of the genus, the red line represents the positive correlation of the genus, the green line represents the negative correlation of the genus, and the line thickness represents the correlation strength. (c) Cooccurrence network analysis in VLPS group. (d) Comparison of gut microbiota ecology between VLPS versus NVLPS groups. Heatmap plot of centrality of nodes (genera), and Venn Diagram shows the number of connections in the two microbial networks. (e) Linear discriminant analysis of taxa enrichment between VLPS versus NVLPS groups. Criteria: Alpha value for the factorial Kruskal-Wallis test among classes < 0.05; Alpha value for the pairwise Wilcoxon test between subclasses <0.05; LDA score for discriminative features > 4. (f) Comparison of α-diversity of Bacteroidetes (Simpson, Shannon index and Observed OTU numbers of Bacteroidetes) between VLPS versus NVLPS groups using the one-way ANOVA. Boxes indicate interquartile range, lines indicate medians, and whiskers represent range.

The PCoA plots did not exhibit clear differences between clusters of antagonist versus agonist long regimen, frozen versus fresh embryos, and low versus high estradiol levels on the HCG day (Supplementary Figures 2a, 3a, and 4a). The positive correlations between the microbiota in the long regimen group were distinctly increased compared to that of antagonist group (Supplementary Figure 2b–d), accordingly, the microbial community of the agonist long regimen group featured a more complicated network, which implicated both treatments might lead to different gut microecology. Although, the frozen group displayed similar networks as fresh group, 84 and 8 connections were specific to frozen and fresh group, respectively. Also, frozen group showed slightly increased positive correlations between microbial relationship compared to fresh group (Supplementary Figure 3b–d). Impressively, the closeness and eigenvector of shared genera were quite similar in the low and high estradiol level groups (Supplementary Figure 4b–d). The LDA enrichment displayed differential associations with the relative abundance of a few taxa (mainly of Bacteroidetes), whereas the α-diversity of Bacteroidetes did not show any difference among specific ART treatment groups (Supplementary Figure 2d–f, 3d–f, and 4d–f)). Taken together, these results implicated different ART treatments might affect microbioal composition and relationship of offspring in different ways.

### Early microbiota composition affects neonatal growth

**Figure 3.**
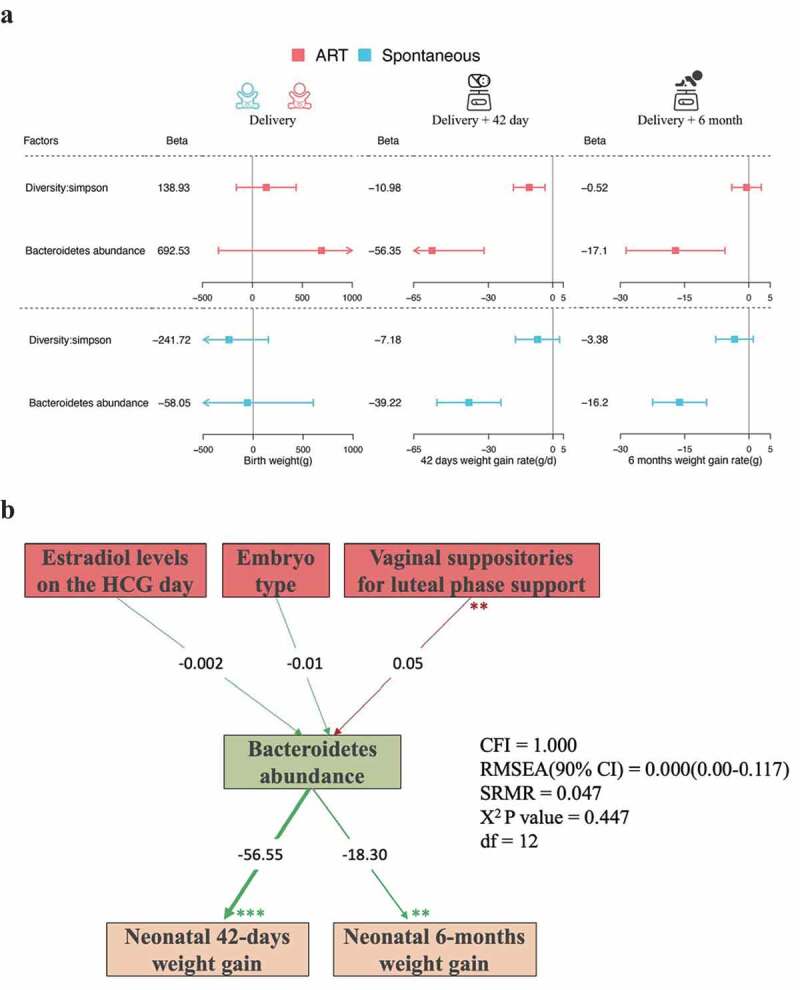
Determinants of neonates gut microbiota composition and the effects of microbiota composition on neonatal growth.

Furthermore, we investigated the associations of early microbiota composition with birth weight, and with 42-day and 6-month weight gain with Fitting linear models. After the adjustment for gestational age, infant gender and maternal pre-pregnancy body mass index (BMI), no association was observed for birth weight with overall α-diversity and Bacteroidetes relative abundance in both ART and spontaneous conception groups (Figure 3a, Table 3). Notably, α-diversity and the relative abundance of Bacteroidetes in ART newborns were inversely associated with their weight gain rate after adjustment for the conventional factors including feeding method. Newborns who had reduced α-diversity (Simpson index) and declined relative abundance of Bacteroidetes showed rapid 42-day weight gaining (Simpson index: β_adj_ = −11.0, 95% CI = −18.3, −3.63, *P* = 4.13 × 10^−3^; Bacteroidetes relative abundance: β_adj_ = −56.4, 95% CI = −80.7, −32.0, *P* = 1.42 × 10^−5^). In spontaneously conceived newborns, the association of 42-day weight gain rate was significantly inverse with Bacteroidetes relative abundance (β_adj_ = −39.2, 95% CI = −54.2, −24.2, *P* = 1.83 × 10^−6^), but was null with overall Simpson index (β_adj_ = −7.20, 95% CI = −17.5, 3.12, *P* = 1.75 × 10^−1^) (Figure 3a, Table 3). We followed up and weighted all infants at their six months of age, α-diversity (Simpson index) and the relative abundance of Bacteroidetes demonstrated inverse but diminished associations with six-month weight gain rate in both ART offspring and infants born after spontaneous conception (Figure 3a, Table 3).

**Table 3.** Association of infant gut microbiota composition with neonatal 42-day and 6-month weight gain rates.

	Birth weight(g)^a^		42 days weight gain rate(g/d)^b^		6 months weight gain rate(g/d)^b^	
	β (95% CI)	*P* value	β (95% CI)	*P* value	β (95% CI)	*P* value
**ART**						
Diversity: Simpson	138.93 (−159.99,437.86)	3.64 × 10^−01^	−10.98 (−18.33,-3.63)	4.13 × 10^−03^	−0.52 (−3.96,2.93)	7.69 × 10^−01^
Bacteroidetes abundance	692.53 (−339.21,1724.28)	1.91 × 10^−01^	−56.35 (−80.67,-32.04)	1.42 × 10^−05^	−17.10 (−28.66,-5.54)	4.54 × 10^−03^
**Spontaneous**						
Diversity: Simpson	−241.72 (−635.71,152.28)	2.33 × 10^−01^	−7.18 (−17.47,3.12)	1.75 × 10^−01^	−3.38 (−7.76,1.01)	1.35 × 10^−01^
Bacteroidetes abundance	−58.05 (−718.95,602.85)	8.64 × 10^−01^	−39.22 (−54.20,-24.24)	1.83 × 10^−06^	−16.20 (−22.50,-9.90)	2.89 × 10^−06^

^a^Linear regression adjusted for gestational age, maternal BMI, and infant gender.

^b^Linear regression adjusted for gestational age, infant gender, birth weight, and feeding method.

(a) Forest plot visualizes the association of meconium microbiota composition with neonatal growth in ART and spontaneous conception groups. Linear regression models were fitted to assess the associations of α-diversity and Bacteroidetes phylum relative abundance with birth weight, weight gain rate during the first 42 days and 6 months of life. (b) Structural Equation Modeling showed that in ART pregnancies, the usage of vaginal suppositories for luteal phase support were independently associated with Bacteroidetes abundance in neonates. Bacteroidetes abundance was inversely associated with weight gain rate. Estimated β coefficients are reported. CFI, comparative fix index; RSMEA, root-mean-square error of approximation; SRMR, standardized root-mean residuals; *P < .05, **P < .01, ***P < .001. Green line represents inverse association; red line represents positive associations.

### Structural equation modeling identified distinct determinants of infant gut microbiota composition in ART-pregnancies

The theoretical framework of how different factors might be associated with gut microbiota composition in ART-conceived neonates was shown as Figure 3b (detailed variable selection was described in Methods section). To evaluate the plausible framework, we performed confirmatory factor analysis (CFA),^18^ which is a variant of structural equation modeling. In our model for the ART conception, the usage of vaginal suppositories for luteal phase support was the main determinant of the abundance of gut Bacteroidetes in the ART neonates (β coefficient = 0.05, *P* < .01; Figure 3b). And the abundance of gut Bacteroidetes was inversely associated with weight gaining rate in the first 42 days and 6 months after birth with β of −56.6 and −18.3, respectively (*P* < .01; Figure 3b).

## Discussion

To the best of our knowledge, we profiled, for the first time, the gut microbiome in newborns conceived after ART across the first four days after birth, which exhibited discrete microbiome composition in the ART offspring, compared with that in the neonates conceived spontaneously. Of particular note, α-diversity (Simpson index) of overall microbiota community and the relative abundance of Bacteroidetes remarkably reduced in the neonates born after ART. Specific ART treatments were the key factors that contributed to gut microbiota composition in offspring. In the investigation of the effect of early microbiota composition on neonatal growth, we found that reduced gut microbiota diversity and declined relative abundance of Bacteroidetes were associated with rapid infant weight gaining, particular at early stage (from birth to 42 days), in both ART and spontaneous conception groups. Together, these results considerably expand upon existing knowledge on gut microbiota composition in neonates born after ART in their very early life. Moreover, we identified specific ART treatments that potentially influenced microbiota composition in offspring and provided evidence for the importance of microbiota composition in neonatal growth.

In agreement with previous studies of infant meconium,^5-7^ our results suggested that factors influencing infant’s intrinsic host microenvironment could drive early life microbiota formation. In our cohort, dominant gut bacteria in the ART and the spontaneous conception groups were both in accordance with previously published findings.^8,19^ Notably, the gut microbiota composition exhibited discrete clusters in infants born by ART versus spontaneous conception birth. We observed declined microbiota diversity in ART neonates, pointing to the less-complex microbiota colonization. In spite of bacterial diversity increases with age and composition gradually resembles that of adults,^8,14^ reduced microbiota α-diversity in meconium may indicate remarkable change of host environment, which could affect infant development after birth.

In addition, the proportion of certain bacterial groups that compose gut microbiome and the rate at which the community stabilizes have been reported to be associated with various health outcomes.^2^ For example, the Bacteroidetes have a very broad metabolic potential, and reduced Bacteroidetes abundance is associated with obesity and irritable bowel syndrome in adults. The gut Bacteroidetes is much less common in infants than in adults, awaiting maturation and gradually resembles that of adults.^20^ The reduced relative abundance of Bacteroidetes in ART birth suggests that their host microenvironment, such as oxygen content, is less suitable for certain microbiome colonization. Estrogen is used in large amounts during ART treatment, which may increase the intrauterine oxygen content,^21^ and inhibit the survival of certain anaerobic bacteria such as Bacteroides. Such changes of microbiome colonization environment may have potential impact on microbiome maturation in later life.

Another key finding of the present study is that specific ART treatments could significantly influence early life formation of neonatal gut microbiome, particularly Bacteroidetes colonization. This supports the hypothesis that intrinsic host microenvironmental factors drive microbiota selection and colonization. Several studies have indicated that gestational age has critical influence on neonatal microbiota development in the gut^6,7^ and in the airway,^5^ which also highlights the importance of host biology. We observed that maternal usage of vaginal suppositories for luteal phase support largely increased the relative abundance of Bacteroidetes in infants, compared with non-vaginal drug usage, and the structure equation indicated that this was the main determinant of gut Bacteroidetes abundance in ART neonates. Whereas the antagonist usage during the ART process, frozen embryo transfer and high estradiol levels on the HCG day reduced gut microbiota Bacteroidetes abundance in offspring. The main component of luteal phase support drug is progesterone, and vaginal suppositories benefits from a first-pass uterine effect, in which endometrial tissue concentrations are typically much greater than would be expected based on serum levels.^22^ It has been reported that oral contraceptive, consisting of progesterone and estrogen, can increase the relative abundance of Bacteroides in dental plague.^23^ In addition, progesterone has the effect of suppressing the increase of intrauterine oxygen content,^21^ which favors Bacteroides colonization. It is biologically plausible that greater progesterone concentrations in uterine microenvironment benefits Bacteroidetes colonization in offspring. To assess the origin of the pioneering microbes in neonate’s first meconium, we integrated the data on the first-pass meconium microbiome in our cohort with maternal microbiota data from a study published by Wang et al.,^24^ and calculated β-diversity (between-samples, Bray-Curtis dissimilarity) between the OTU profiles of neonates gut microbiota and those of pregnant women’s oral, vaginal and intestinal microbiota. In the principal coordinate analysis (PCoA) of the similarity of microbiome clusters, we found that meconium microbiome in the infants born after ART and spontaneous conception were both greatly overlapped with vaginal and intestinal microbiome of pregnant women (Supplementary Figure 5). These suggested that most of the early colonizers in infants are derived from their mothers, and women’s vagina and gut microbes might be the first colonizers. Our findings demonstrated that vertical mother-infant transmission was less frequent for Bacteroidetes in the ART infants, while maternal usage of vaginal suppositories for luteal phase support can compensate the reduced abundance of this important intestinal microbe.

Evidence is mounting that the early-in-life microbial population quite likely influences later-in-life host biology, health and development.^25-28^ Thus, together with the profile of neonates gut microbiome, we also followed up infants for their growth to six months after birth, and underscored the role of gut microbiota in weight gain during early infancy. It is acknowledged that rapid weight gain in infancy is a risk factor for later development of obesity.^29,30^ Human gut microbiota, through a symbiotic relationship with the host, has been implicated in maintaining metabolic homeostasis. Dysbiosis has been linked to obesity in adults, with reduced bacterial diversity, lower proportion of Bacteroidetes and a high F/B ratio being associated with greater risk of obese.^15-17,27^ In agreement with these findings, our data demonstrated that reduced gut microbiota diversity and declined relative abundance of Bacteroidetes in infants were associated with their rapid weight gaining in the first six months of life. Animal models of obesity suggested that Bacteroides species might play a protective role in against obesity through increasing energy harvest from diet and altering fatty acid metabolism and composition in adipose tissue and liver.^31^ White et al. investigated early infant gut microbiota in relation to the growth in the first six months of life and found the non-detection of Bacteroides species at day 30 was associated with rapid growth.^28^ Our study suggests that the microbiome acquired at a very early stage of life is poised to influence upcoming infant growth and development.

The present study has some notable strengths. The prospective study design and postpartum follow-up allowed examination of the associations of microbiota composition with prospective maternal and clinical factors as well as, with subsequent infant growth and development. In addition, it included both ART offspring and neonates born following spontaneous conception, and all follow-up and data collection followed the same procedures without knowledge of the conception type. Our findings elucidate the distinction of gut microbiota composition in ART versus spontaneous conception but a similar effect of microbiota composition on neonatal growth.

Some potential limitations of the present study also merit discussion. First, maternal samples for microbiome analysis were not collected during their gestations, and thus we were not able to determine whether maternal and early life factors shape microbiota colonization in the fetus through influencing maternal origin microbiome. Nevertheless, our cluster analysis of microbiota from diverse sources demonstrated a great similarity in microbiome composition between infant meconium and samples from women’s vagina and intestine. Secondly, in the present study, we collected data from infants when they met their six months of age. The relatively short follow-up time may have limited the investigation on other health-related outcomes. The associations of infant microbiota composition with diseases and wellbeing need to be further examined in future studies.

Collectively, in this longitudinal and prospective study, we detected a distinct gut microbiome composition between neonates born to ART and spontaneous conception. We observed a significantly reduced microbiota diversity and a declined relative abundance of Bacteroidetes in the meconium samples from the neonates born after ART, which may shed light upon previously identified differences between ART offspring and children born following spontaneous conception, regarding their health and development outcomes. The microbiota composition of neonates born by ART was largely driven by specific ART treatments, further emphasizing the importance of fetus intrinsic host microenvironment in the microbial colonization and formation in very early life. Our follow-up data on neonatal growth indicated the critical impact of infant gut microbiota composition on rapid weight gaining in their early life, which provides evidence linking early life microbiome with obesity in later life. Although our current results reveal a correlation between infants’ weight at the 6 months and the meconium microbe, a long-term longitudinal study is needed to validate the conclusion that infant weight is pre-determined meconium microbiome.

## Methods

### Study population

The participants were from the China National Birth Cohort (CNBC) Study, which is a prospective and longitudinal study of both assisted reproductive technology (ART) pregnancies and spontaneous pregnancies. The CNBC Study was mainly designed for comprehensively comparing the birth outcomes of ART and spontaneous-conceived pregnancies and systematically evaluating the environmental and genetics factors that may influence birth outcomes. In the present study, we enrolled 228 healthy singleton newborns who were born to a cohort of women after 35 gestational weeks, and had first-pass meconium samples collected between August 1, 2017 to July 31, 2018 from Nanjing Maternity and Child Health Care Hospital and Suzhou Municipal Hospital in China. Newborns who were diagnosed of congenital abnormalities or their mothers had significant pregnancy complications were not recruited in this study. We further excluded newborns from this study if key maternal (e.g., ART treatments, BMI) or infant (e.g., birth weight, 42-day weight) data were missing (*n* = 19). In total, 209 newborns were included in the analytical population, among which 118 were born after ART and 91 were born after spontaneous conception.

All women participated in the CNBC Study were recruited at their early pregnancy (spontaneous pregnancies) or before ART procedures (ART pregnancies), and were followed up during antenatal, perinatal and postnatal periods. Spontaneous pregnancy families were recruited at their first antenatal visits (Enrollment Visit: 8–14 weeks of gestation), while ART families were recruited before the first egg retrieval cycle at ART clinics (Enrollment Visit: 1 week before the ART treatment cycle). ART-treated and spontaneous-conceived women completed standardized and structured questionnaires by face-to-face interview to collect their demographic information and reproductive history. During parturition (Delivery Visit), pregnancy outcomes were collected from the medical records and neonates’ anthropometric parameters were measured. All children visited the clinic for health examination and anthropometric measurement approximately 42 days after the birth and then 6 months of age. This study was approved by the institutional review board of Nanjing Medical University (FWA00001501). Written informed consent was obtained for participation from all families.

### Sample collection and processing

In addition to the first-pass meconium, fecal samples were also collected in the following three days after birth from each newborns during hospitalization if available. On the second, third and fourth day after birth, we collected 65/56, 43/41, and 12/21 fecal samples from ART-/spontaneous-neonates, respectively. Samples were refrigerated immediately after collection and stored at −80°C until analysis.

### DNA extraction and 16S rRNA sequencing

Genomic DNA was extracted from 180 to 220 mg of stool using the QIAamp Fast DNA Stool Mini Kit (Qiagen, Hilden, Germany) following the manufacturer’s instructions.^32^ The V3-V4 hypervariable region of the bacterial 16S rRNA gene was amplified by PCR using the universal bacterial 16S rRNA gene PCR amplicon primers (forward primer, 338 F: 5ʹ-ACTCCTACGGGAGGCAGCAG-3ʹ; reverse primer, 806 R: 5ʹ-GGACTACHVGGGTWTCTAAT-3ʹ)^33^ combined with adapter sequences and barcode sequences. PCR amplification was performed in a total volume of 20 ul, which contained 10 ng template DNA, 4ul 5× FastPfu Buffer, 2ul 2.5 Mm dNTPs, 0.8ul forward primer (5 uM), 0.8 ul reverse primer (5 uM), 0.4 ul FastPfu Polymerase, 0.2ul BSA and the rest are ddH2O. PCR amplification consisted of an initial denaturation step for 3 min at 95°C, followed by 27 cycles of denaturation for 30 s at 95°C, annealing for 30 s at 53°C and an extension step for 45 s at 72°C, and then for 10 min at 72°C with a completion step at 10°C. Mixed PCR products were purified using the GeneJET Gel Extraction Kit (Thermo Scientific, USA) following the manufacturer’s instructions. High-throughput sequencing analysis of bacterial rRNA genes was performed on the purified, pooled sample using the Illumina Miseq platform (2 × 300 paired ends) at Majorbio, Shanghai, China. Paired-end reads from the original DNA fragments were merged using FLASH (version 1.2.11).^34^ To rule out potential contaminants, 4 background samples collected from the hospital environment were also included in PCR and 16S sequencing. Paired-end reads (tags) were assigned to each sample according to the unique barcodes and raw tags were quality controlled by Trimmomatic (version 0.39).^35^ High quality tag sequences were obtained by truncating the first low quality (50 base position window, mean Phred score < 20) window start base sites, filtering out the tags of continuous high-quality base length less than 50 base position or the tags contain N base. According to the overlap relationship between tags, pairwise reads are merged into a sequence, the minimum overlap length is 10 base position, the maximum mismatch ratio allowed for the overlap region is 0.2. Samples are differentiated based on barcode and primer sequences, the allowed mismatch base number for barcode is 0 and 2 for primer. Operational Taxonomic Units (OTUs) were generated at 97% identity threshold using QIIME (version 1.9.1) open-source bioinformatics pipeline.^36^ OTUs < 20 reads across the entire dataset were removed. The seed sequences of each OTU were chosen for taxonomic classification against the Greengene reference sequences (gg_13_8_otus)^37^ by the UCLUST taxonomy assigner. After all, the meconium microbiome profiles revealed a bacterial community composed of 14,437 OTUs, which then can be assigned to 64 unique phyla. The 16S rRNA gene sequencing datasets used in this study are stored in the National Center for Biotechnology Information (NCBI) Sequence Read Archive: https://dataview.ncbi.nlm.nih.gov/object/PRJNA574003?reviewer=h3rb2soaipmdhn6m1cesqn1000.

### Ecological parameters

The total diversity in the OTU profile was decomposed into α-diversity (within-sample) and β-diversity (between-samples) according to the method proposed by Rao^38^ and Bray.^39^ α-diversity was assessed by Simpson (value range from 0 to 1, the greater the value, the higher the diversity), Shannon Index and observed OTU numbers. β-diversity was estimated using Principal Coordinate Analysis (PCoA) with vegdist function on Bray-Curtis dissimilarity matrix (vegan package^40^). Dissimilarity (β-diversity) of group was assessed by permutational ANOVA (PERMANOVA) with Adonis function (vegan package). Differentially abundant microbiota taxa between ART and spontaneous-conceived groups were identified at phylum and genus levels using MaAsLin (Multivariate Analysis by Linear Models) with Maaslin2 function (Maaslin2 package, the next generation of MaAsLin).^41^ The MaAslin boosting step was turned off to ensure all independent variables were taken into account. Considering that the dependent variables includes multiple different microbiota taxa, at each level, taxa with an FDR (*Q*-value) < 0.2 as differentially abundant microbiota taxa.

### Co-occurrence network analysis

To understand the correlations among different genera, we constructed co-occurrence network based on the 16S rRNA data. We first calculated the relative abundance of each genus in total 209 first-pass meconium, and only the genera with relative abundance > 0 in more than half of the samples were used in the construction of all the 10 co-occurrence networks of genera. Next, we grouped the first-pass meconium samples according to different characteristics (e.g., ART or spontaneous group), and then calculated the correlations between each two of the selected genera within each group using Spearman’s correlation coefficient analysis. The significantly correlated genus (false discovery rate, FDR < 0.05) were visualized by Cytoscape (v3.6.1). The differences between any two network structures were compared by node closeness centralities and shared correlations.^42^ The shared correlations between two groups were defined the edges with the same nodes in two co-occurrence networks. Closeness centralities of the nodes was calculated by Cytoscape (v3.6.1) to measure node centralities in each network. The difference of bacterial correlations in any two groups was quantified by counting the same and different genus correlations across sample groups. The results were visualized by the R (version 3.5.1) (VennDiagram^43^ and gplots package^44^).

### Concordance of the microbiota between sample

We used the β-diversity (Bray-Curtis dissimilarity) to measure the dissimilarity between microbiomes in the first-pass meconium and those in the later fecal samples, based on the relative abundance of OTUs.

### Association analysis

Associations of α-diversity with maternal, infant and ART processing factors were assessed by linear regression in multivariate analysis adjusting for modes of delivery, gestational age and infant gender. Considering that the distribution of Bacteroidetes abundance in the meconium is a positively skewed distribution rich in zero. Associations of Bacteroidetes abundance with maternal, infant and ART processing factors were assessed by multivariate Tobit regression analysis adjusting for mode of delivery, gestational age and infant gender.^45^ Associations of infant birth weight with microbial α-diversity and Bacteroidetes relative abundance in first-pass meconium were assessed by linear regression adjusting for gestational age, infant gender and maternal pre-pregnancy BMI. Infant weight gain was assessed as average daily weight gain from birth to 42 days (range from 28 to 50 days) after birth and to 6 months (range from 23 to 28 weeks) of age. Associations of weight gain with α-diversity and Bacteroidetes relative abundance were assessed by linear regression adjusting for gestational age, infant gender, birth weight, and feeding method.

### Structural equation modeling

Structural equation modeling (SEM) was conducted using lavaan package^46^ and path diagrams were visualized using semPlot package. Variable selection was informed by the results of tobit models explained above. Model fit was assessed by χ2 test, the comparative fix index (CFI), root mean square error of approximation (RSMEA) and its 90% confidence interval (CI), and the standardized root mean residuals (SRMR). Nonsignificant χ2 test, CFI > 0.9, RSMEA < 0.05 and SRMR < 0.08 were considered as indications of good model fit.^18^

### Linear discriminant analysis

Taxa enrichment by specific ART treatments was assessed by linear discriminant analysis (LDA) effect size (LEfSe) with default parameters and LDA score threshold of four.^47^

## Supplementary Material

Supplemental Material
