# Supplementary material for: Alternations of gut microbiota composition in neonates conceived by assisted reproductive technology and its relation to infant growth
Source: Gut Microbes. 2020 Aug 4;12(1):1794466. doi: 10.1080/19490976.2020.1794466 (PMC7524295; doi:10.1080/19490976.2020.1794466)
Supplement: Supplemental Material [file KGMI_A_1794466_SM4843.pdf]

Supporting information

Alternations of gut microbiota composition in neonates conceived by assisted reproductive technology and its relation to infant growth

Qun Lu, Yuan Lin, Ting Chen, Hong Lv, Feiyang Diao, Cong Liu, Meijuan Peng, Xiufeng Ling, Hong Li, Yun Wang, Yongyue Wei, Jiangbo Du, Guangfu Jin, Yankai Xia, Hongxia Ma, Xingyin Liu, Hongbing Shen, Zhibin Hu

Supplementary Fig. 1. The first meconium microbiome exhibit discrete composition between ART- and spontaneous-conceived neonates.

A. Co-occurrence network analysis in ART conception group. Each node represents a genus, nodes of the same color belong to the same phylum, the significantly correlated genera (FDR < 0.05) were visualized. The node size represents the relative abundance of the genus, the red line represents the positive correlation of the genus, the green line represents the negative correlation of the genus, and the line thickness represents the correlation strength.

B. Co-occurrence network analysis in spontaneous conception group.

C. Comparison between two groups' gut microbiota ecology. Heatmap plot of centrality of nodes (genera), and Venn Diagram shows the number of connections in the two microbial networks.

D. Log (relative abundance of Firmicutes/relative abundance of Bacteroidetes) between ART and spontaneous conception groups (and between ART caesarean section and spontaneous caesarean section groups; C/S, caesarean section). Boxes indicate interquartile range, lines indicate medians, and whiskers represent range.

E. Intra-individual β-diversity (Bray-Curtis dissimilarity) between microbiomes in the first-pass meconium and those in the later fecal samples in ART and spontaneous conception groups. Boxes indicate interquartile range, lines indicate medians, and whiskers represent range.

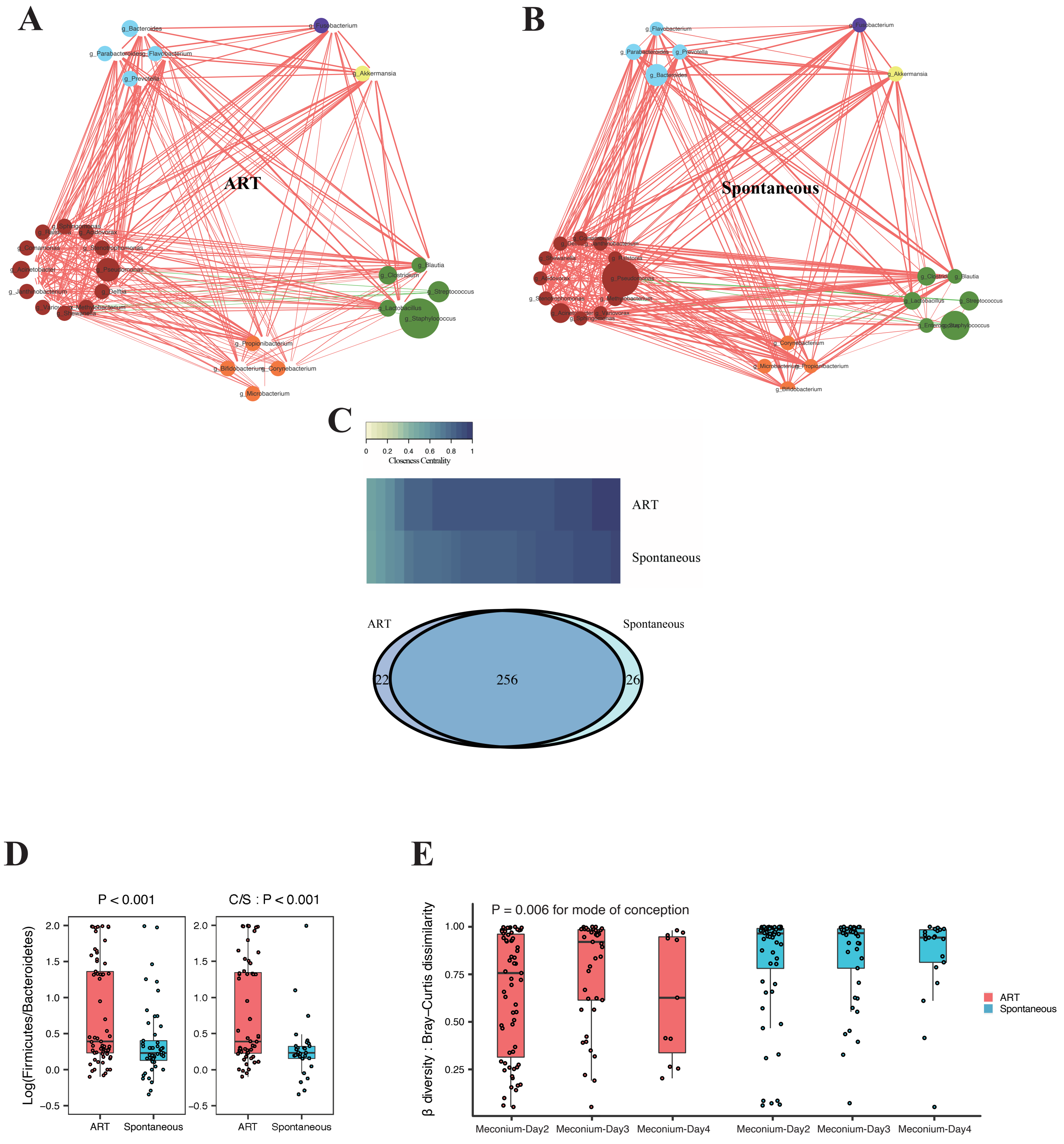
A
B
ART
Spontaneous
C
Closeness Centrality
0
0.2
0.4
0.6
0.8
1
ART
Spontaneous
ART
Spontaneous
22
256
26
D
E
P < 0.001
C/S : P < 0.001
Log(Firmicutes/Bacteroidetes)
ART
Spontaneous
P = 0.006 for mode of conception
β diversity : Bray–Curtis dissimilarity
Meconium-Day2
Meconium-Day3
Meconium-Day4
ART
Spontaneous

Supplementary Fig. 2. Regimen of ovulation was in relation to gut Bacteroidetes relative abundance in the neonates conceived by ART.

A. Comparison of β diversity between agonist long regimen and antagonist regimen groups using PERMANOVA.

B. Co-occurrence network analysis in agonist long regimen group.

C. Co-occurrence network analysis in antagonist regimen group.

D. Comparison between two groups' gut microbiota ecology. Heatmap plot of centrality of nodes (genera), and Venn Diagram shows the number of connections in the two microbial networks.

E. Linear discriminant analysis of taxa enrichment between agonist long regimen and antagonist regimen groups. Criteria: Alpha value for the factorial Kruskal-Wallis test among classes < 0.05; Alpha value for the pairwise Wilcoxon test between subclasses < 0.05; LDA score for discriminative features > 4.

F. Comparison of α-diversity of Bacteroidetes (Simpson, Shannon index and Observed OTU numbers of Bacteroidetes) between agonist long regimen and antagonist regimen groups.

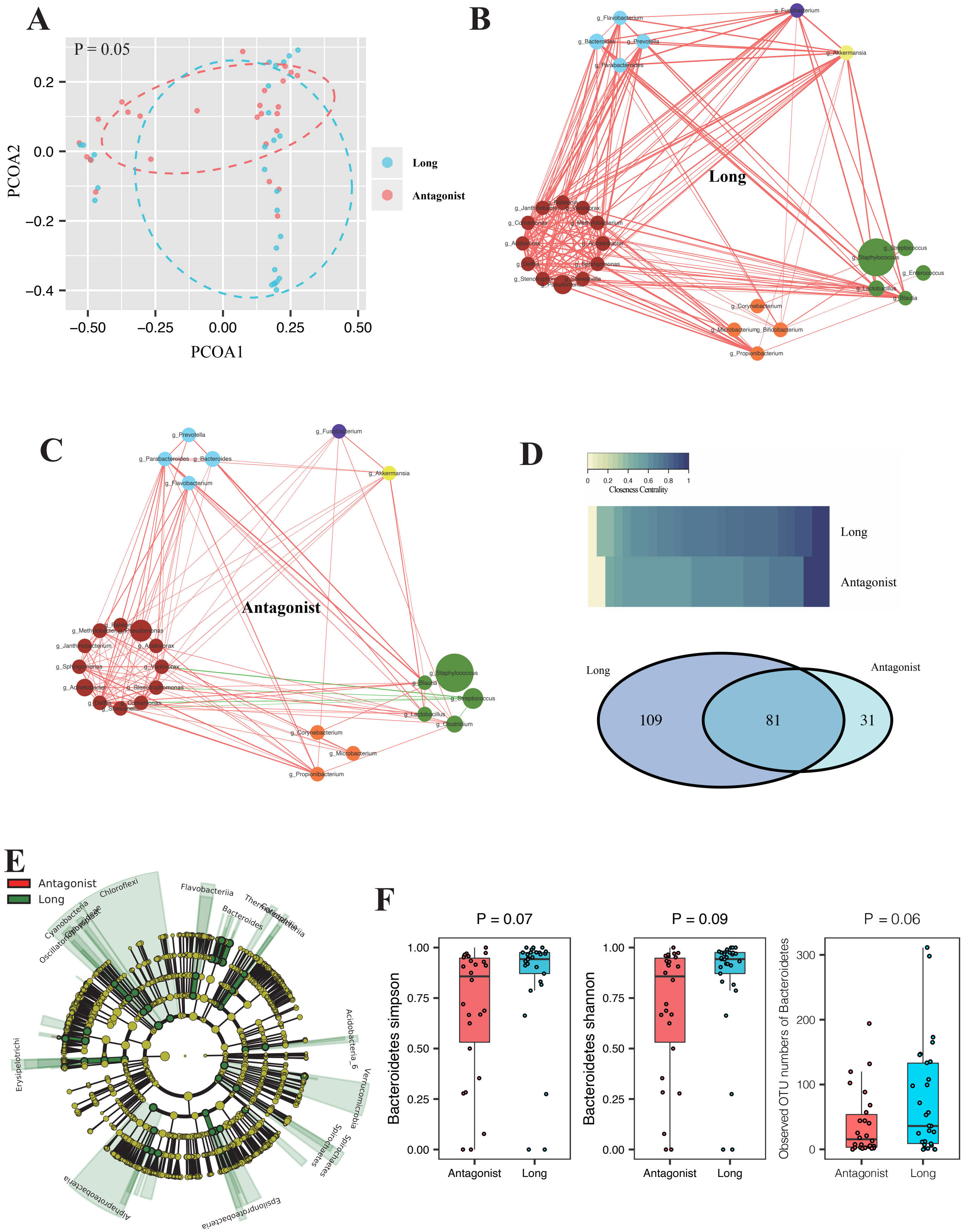
A
P = 0.05
PCOA1
PCOA2
Long
Antagonist
B
Long
g_Flavobacterium
g_Fusobacterium
g_Bacteroides
g_Prevotella
g_Akkermansia
g_Parabacteroides
g_Staphylococcus
g_Streptococcus
g_Enterococcus
g_Lactobacillus
g_Blautia
g_Corynebacterium
g_Microbacterium
g_Bifidobacterium
g_Propionibacterium
C
Antagonist
g_Prevotella
g_Fusobacterium
g_Parabacteroides
g_Bacteroides
g_Akkermansia
g_Flavobacterium
g_Pseudomonas
g_Janthinobacterium
g_Acidovorax
g_Sphingomonas
g_Acinetobacter
g_Stenotrophomonas
g_Comamonas
g_Staphylococcus
g_Blautia
g_Streptococcus
g_Lactobacillus
g_Clostridium
g_Corynebacterium
g_Microbacterium
g_Propionibacterium
D
Closeness Centrality
Long
Antagonist
109
81
31
E
Antagonist
Long
Chloroflexi
Flavobacteriia
Bacteroides
Cyanobacteria
Chloroplast
Erysipelotrichi
Acidobacteria_6
Verrucomicrobia
Spirochaetes
Alphaproteobacteria
Epsilonproteobacteria
F
P = 0.07
P = 0.09
P = 0.06
Bacteroidetes simpson
Bacteroidetes shannon
Observed OTU numbers of Bacteroidetes
Antagonist
Long

Supplementary Fig. 3. Type of embryo was in relation to the relative abundance of gut Bacteroidetes in the neonates conceived by ART.

A. Comparison of β diversity between frozen embryo transfer and fresh embryo transfer groups using PERMANOVA.

B. Co-occurrence network analysis in frozen embryo transfer group.

C. Co-occurrence network analysis in fresh embryo transfer group.

D. Comparison between two groups' gut microbiota ecology. Heatmap plot of centrality of nodes (genera), and Venn Diagram shows the number of connections in the two microbial networks.

E. Linear discriminant analysis of taxa enrichment between frozen embryo transfer and fresh embryo transfer groups. Criteria: Alpha value for the factorial Kruskal-Wallis test among classes < 0.05; Alpha value for the pairwise Wilcoxon test between subclasses < 0.05; LDA score for discriminative features > 4.

F. Comparison of α-diversity of Bacteroidetes (Simpson, Shannon index and Observed OTU numbers of Bacteroidetes) between frozen embryo transfer and fresh embryo transfer groups.

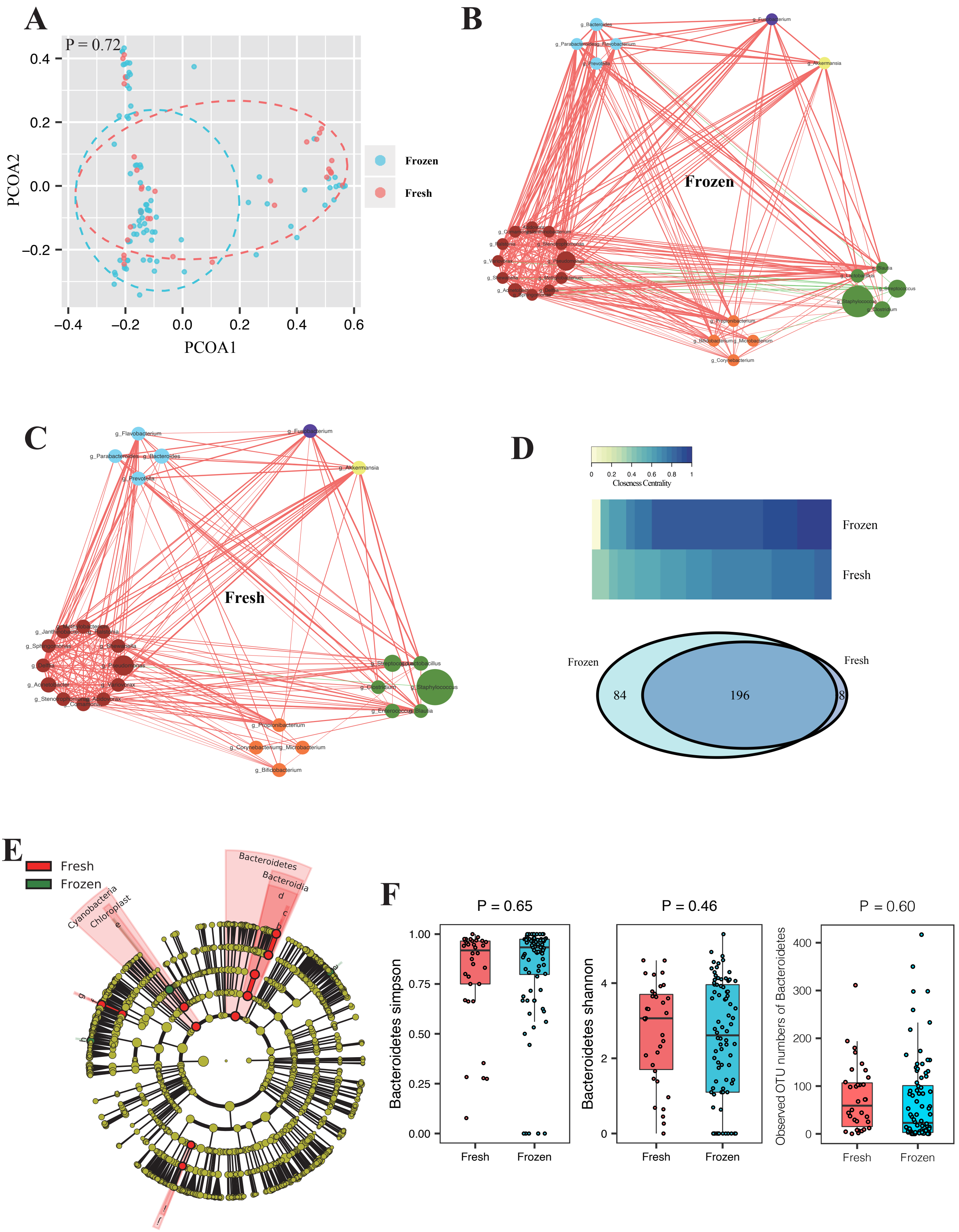
A
P = 0.72
PCOA2
PCOA1
Frozen
Fresh
B
Frozen
C
Fresh
g_Flavobacterium
g_Fusobacterium
g_Parabacteroides
g_Bacteroides
g_Akkermansia
g_Prevotella
g_Propionibacterium
g_Corynebacterium
g_Microbacterium
g_Bifidobacterium
g_Staphylococcus
D
Closeness Centrality
Frozen
Fresh
84
196
8
E
Fresh
Frozen
Cyanobacteria
Chloroplast
Bacteroidetes
Bacteroidia
F
P = 0.65
P = 0.46
P = 0.60
Bacteroidetes simpson
Bacteroidetes shannon
Observed OTU numbers of Bacteroidetes
Fresh
Frozen

Supplementary Fig. 4. Estradiol level was in relation to the relative abundance of gut Bacteroidetes in the neonates conceived by ART.

A. Comparison of β diversity between high HCG day estradiol level and low HCG day estradiol level groups using PERMANOVA.

B. Co-occurrence network analysis in high HCG day estradiol level group.

C. Co-occurrence network analysis in low HCG day estradiol level group.

D. Comparison between two groups' gut microbiota ecology. Heatmap plot of centrality of nodes (genera), and Venn Diagram shows the number of connections in the two microbial networks.

E. Linear discriminant analysis of taxa enrichment between high HCG day estradiol level and low HCG day estradiol level groups. Criteria: Alpha value for the factorial Kruskal-Wallis test among classes < 0.05; Alpha value for the pairwise Wilcoxon test between subclasses < 0.05; LDA score for discriminative features > 4.

F. Comparison of α-diversity of Bacteroidetes (Simpson, Shannon index and Observed OTU numbers of Bacteroidetes) between high HCG day estradiol level and low HCG day estradiol level groups.

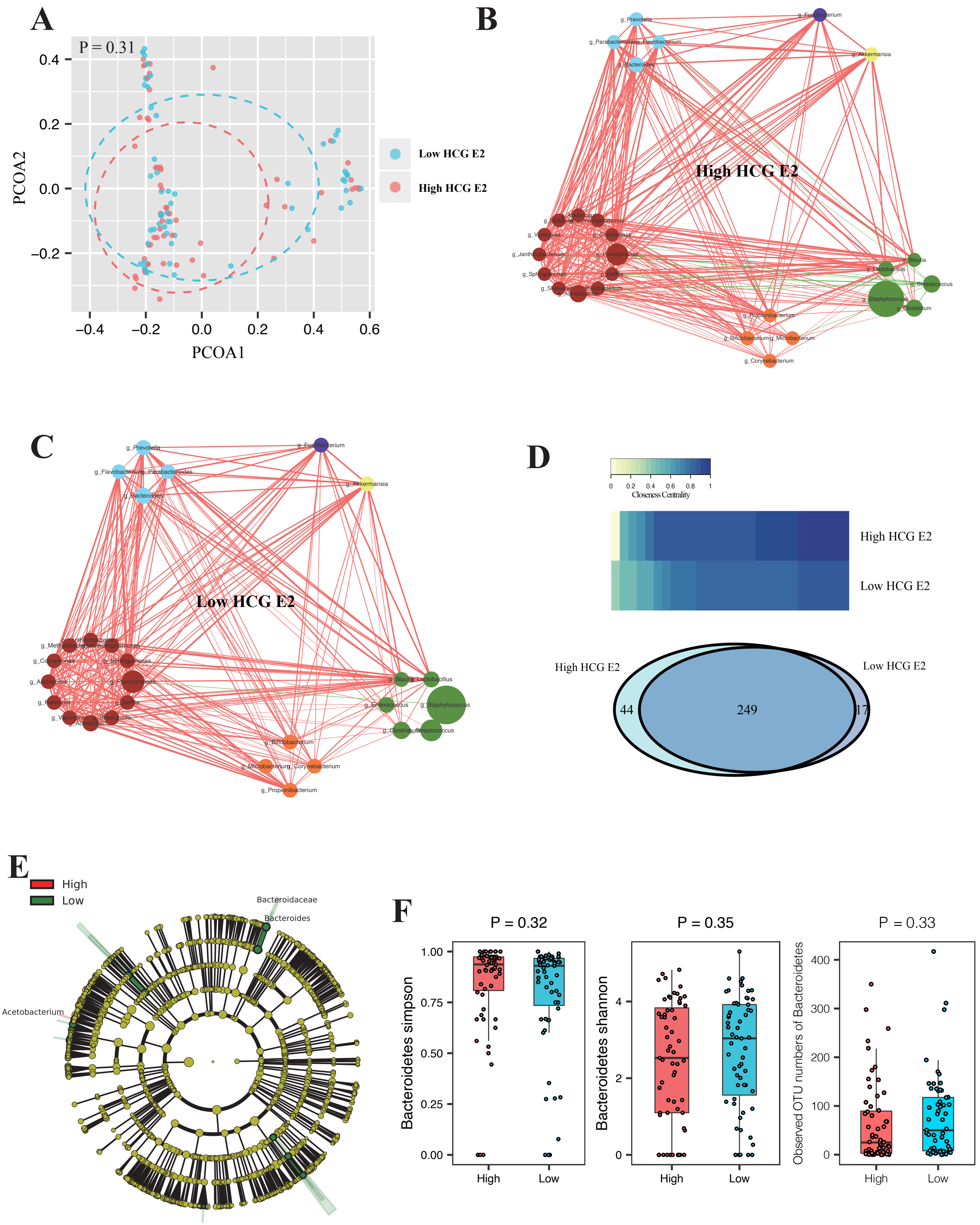
A
P = 0.31
PCOA1
PCOA2
Low HCG E2
High HCG E2
B
High HCG E2
g_Prevotella
g_Fusobacterium
g_Akkermansia
g_Bacteroides
g_Flavobacterium
g_Blautia
g_Lactobacillus
g_Streptococcus
g_Staphylococcus
g_Clostridium
g_Propionibacterium
g_Bifidobacterium
g_Microbacterium
g_Corynebacterium
C
Low HCG E2
g_Prevotella
g_Fusobacterium
g_Flavobacterium
g_Parabacteroides
g_Akkermansia
g_Bacteroides
g_Blautia
g_Lactobacillus
g_Enterococcus
g_Staphylococcus
g_Streptococcus
g_Bifidobacterium
g_Corynebacterium
g_Propionibacterium
D
Closeness Centrality
High HCG E2
Low HCG E2
44
249
17
E
High
Low
Bacteroidaceae
Bacteroides
Acetobacterium
F
P = 0.32
P = 0.35
P = 0.33
Bacteroidetes simpson
Bacteroidetes shannon
Observed OTU numbers of Bacteroidetes
High
Low

Supplementary Fig. 5. Principal Coordinate Analysis (PCoA) for microbiota in the samples collected from different body sites of pregnant women from the study by Wang et al. and in the meconium samples from the neonates born via ART and following spontaneous conception in our cohort.

A. PCoA of the Bray-Curtis distances of oral, intestinal and vaginal microbiota from the pregnant women, and meconium microbiota from the neonates born via ART.

B. PCoA of the Bray-Curtis distances of oral, intestinal and vaginal microbiota from the pregnant women, and meconium microbiota from the neonates born following spontaneous conception.

C. PCoA of the Bray-Curtis distances of oral, intestinal and vaginal microbiota from the pregnant women without GDM, and meconium microbiota from the neonates born via ART.

D. PCoA of the Bray-Curtis distances of oral, intestinal and vaginal microbiota from the pregnant women without GDM, and meconium microbiota from the neonates born following spontaneous conception.

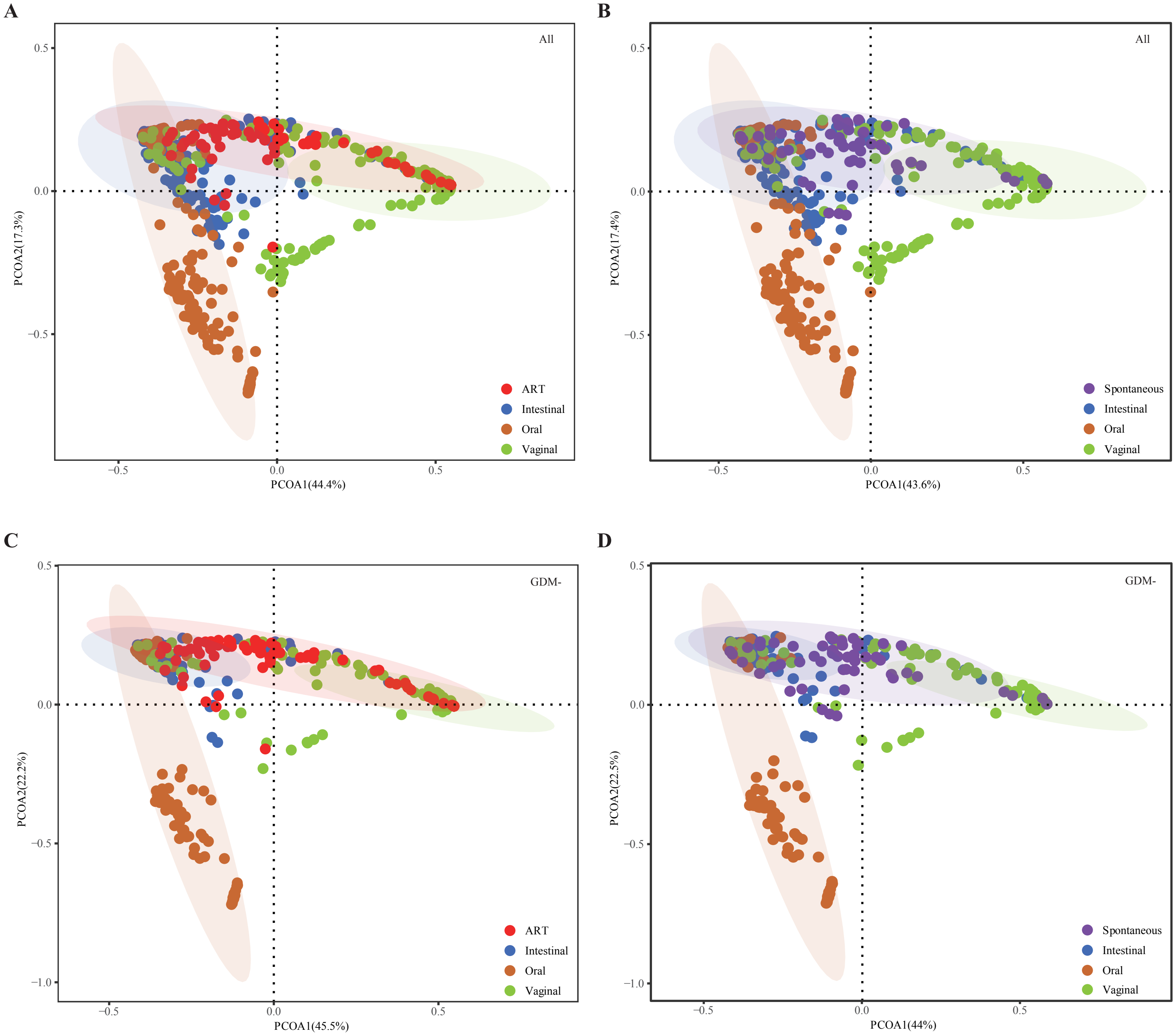

A
All
PCOA1(44.4%)
PCOA2(17.3%)
ART
Intestinal
Oral
Vaginal
B
All
PCOA1(43.6%)
PCOA2(17.4%)
Spontaneous
Intestinal
Oral
Vaginal
C
GDM-
PCOA1(45.5%)
PCOA2(22.2%)
ART
Intestinal
Oral
Vaginal
D
GDM-
PCOA1(44%)
PCOA2(22.5%)
Spontaneous
Intestinal
Oral
Vaginal

Supplementary Table 1. Differentially abundant core bacterial phyla and genera (mean relative abundance >1%) in the first-pass meconium samples from neonates conceived by ART and after spontaneous conception

Metadata	Genus	Value	Coefficient	P-value	Q-value
Conception group	Acinetobacter	Spontaneous	-0.0033	8.68×10^{-01}	9.41×10^{-01}
Conception group	Bacteroides	Spontaneous	0.1351	1.28×10^{-05}	8.19×10^{-04}
Conception group	Clostridium	Spontaneous	-0.0155	4.54×10^{-01}	7.37×10^{-01}
Conception group	Enterococcus	Spontaneous	-0.0120	4.76×10^{-01}	7.42×10^{-01}
Conception group	Lactobacillus	Spontaneous	0.0377	9.97×10^{-02}	5.76×10^{-01}
Conception group	Pseudomonas	Spontaneous	-0.0009	9.80×10^{-01}	9.81×10^{-01}
Conception group	Staphylococcus	Spontaneous	-0.1188	1.05×10^{-02}	1.34×10^{-01}
Conception group	Streptococcus	Spontaneous	-0.0455	1.96×10^{-01}	6.94×10^{-01}
Maternal age (years)	Acinetobacter	/	0.0023	7.90×10^{-01}	9.21×10^{-01}
Maternal age (years)	Bacteroides	/	0.0120	3.54×10^{-01}	7.26×10^{-01}
Maternal age (years)	Clostridium	/	-0.0061	4.88×10^{-01}	7.42×10^{-01}
Maternal age (years)	Enterococcus	/	-0.0002	9.81×10^{-01}	9.81×10^{-01}
Maternal age (years)	Lactobacillus	/	0.0047	6.28×10^{-01}	8.04×10^{-01}
Maternal age (years)	Pseudomonas	/	0.0011	9.46×10^{-01}	9.81×10^{-01}
Maternal age (years)	Staphylococcus	/	0.0203	3.02×10^{-01}	6.99×10^{-01}
Maternal age (years)	Streptococcus	/	-0.0285	5.76×10^{-02}	5.13×10^{-01}
Infant gender	Acinetobacter	Male	-0.0043	7.91×10^{-01}	9.21×10^{-01}
Infant gender	Bacteroides	Male	0.0347	1.66×10^{-01}	6.24×10^{-01}
Infant gender	Clostridium	Male	-0.0242	1.58×10^{-01}	6.24×10^{-01}
Infant gender	Enterococcus	Male	0.0154	2.67×10^{-01}	6.99×10^{-01}
Infant gender	Lactobacillus	Male	0.0193	3.08×10^{-01}	6.99×10^{-01}
Infant gender	Pseudomonas	Male	-0.0386	2.06×10^{-01}	6.94×10^{-01}
Infant gender	Staphylococcus	Male	-0.0045	9.05×10^{-01}	9.65×10^{-01}
Infant gender	Streptococcus	Male	0.0291	3.17×10^{-01}	6.99×10^{-01}
Gestational age (weeks)	Acinetobacter	/	0.0083	3.63×10^{-01}	7.26×10^{-01}

Gestational age (weeks)	Bacteroides	/	-0.0250	7.21×10^{-02}	5.13×10^{-01}
Gestational age (weeks)	Clostridium	/	0.0114	2.27×10^{-01}	6.99×10^{-01}
Gestational age (weeks)	Enterococcus	/	-0.0082	2.83×10^{-01}	6.99×10^{-01}
Gestational age (weeks)	Lactobacillus	/	0.0084	4.23×10^{-01}	7.37×10^{-01}
Gestational age (weeks)	Pseudomonas	/	0.0061	7.15×10^{-01}	8.98×10^{-01}
Gestational age (weeks)	Staphylococcus	/	0.0065	7.57×10^{-01}	9.21×10^{-01}
Gestational age (weeks)	Streptococcus	/	-0.0044	7.84×10^{-01}	9.21×10^{-01}
Gravidity	Acinetobacter	> 0	0.0112	5.10×10^{-01}	7.42×10^{-01}
Gravidity	Bacteroides	> 0	-0.0194	4.54×10^{-01}	7.37×10^{-01}
Gravidity	Clostridium	> 0	-0.0130	4.61×10^{-01}	7.37×10^{-01}
Gravidity	Enterococcus	> 0	0.0096	5.02×10^{-01}	7.42×10^{-01}
Gravidity	Lactobacillus	> 0	-0.0178	3.61×10^{-01}	7.26×10^{-01}
Gravidity	Pseudomonas	> 0	-0.0183	5.61×10^{-01}	7.80×10^{-01}
Gravidity	Staphylococcus	> 0	0.0066	8.67×10^{-01}	9.41×10^{-01}
Gravidity	Streptococcus	> 0	0.0186	5.35×10^{-01}	7.61×10^{-01}
Mode of delivery	Acinetobacter	Vaginal	0.0151	4.51×10^{-01}	7.37×10^{-01}
Mode of delivery	Bacteroides	Vaginal	-0.1064	5.91×10^{-04}	1.64×10^{-02}
Mode of delivery	Clostridium	Vaginal	-0.0107	6.09×10^{-01}	7.95×10^{-01}
Mode of delivery	Enterococcus	Vaginal	-0.0092	5.87×10^{-01}	7.89×10^{-01}
Mode of delivery	Lactobacillus	Vaginal	-0.0787	7.67×10^{-04}	1.64×10^{-02}
Mode of delivery	Pseudomonas	Vaginal	0.1219	1.21×10^{-03}	1.94×10^{-02}
Mode of delivery	Staphylococcus	Vaginal	0.0379	4.15×10^{-01}	7.37×10^{-01}
Mode of delivery	Streptococcus	Vaginal	-0.0366	3.01×10^{-01}	6.99×10^{-01}
Maternal pre-pregnancy BMI (kg/m^2)	Acinetobacter	/	-0.0097	2.46×10^{-01}	6.99×10^{-01}
Maternal pre-pregnancy BMI (kg/m^2)	Bacteroides	/	-0.0110	3.87×10^{-01}	7.37×10^{-01}
Maternal pre-pregnancy BMI (kg/$m^{2)}$	Clostridium	/	-0.0089	3.10×10^{-01}	6.99×10^{-01}
Maternal pre-pregnancy BMI (kg/$m^{2)}$	Enterococcus	/	-0.0074	2.97×10^{-01}	6.99×10^{-01}
Maternal pre-pregnancy BMI (kg/$m^{2)}$	Lactobacillus	/	0.0168	8.09×10^{-02}	5.18×10^{-01}

Maternal pre-pregnancy BMI (kg/m^{2})	Pseudomonas	/	0.0290	6.21×10^{-02}	5.13×10^{-01}
Maternal pre-pregnancy BMI (kg/m^{2})	Staphylococcus	/	-0.0104	5.91×10^{-01}	7.89×10^{-01}
Maternal pre-pregnancy BMI (kg/m^{2})	Streptococcus	/	0.0213	1.50×10^{-01}	6.24×10^{-01}
Passive smoking exposure during pregnancy	Acinetobacter	Yes	0.0007	9.67×10^{-01}	9.81×10^{-01}
Passive smoking exposure during pregnancy	Bacteroides	Yes	-0.0402	1.17×10^{-01}	5.76×10^{-01}
Passive smoking exposure during pregnancy	Clostridium	Yes	-0.0041	8.16×10^{-01}	9.32×10^{-01}
Passive smoking exposure during pregnancy	Enterococcus	Yes	0.0205	1.50×10^{-01}	6.24×10^{-01}
Passive smoking exposure during pregnancy	Lactobacillus	Yes	-0.0351	7.03×10^{-02}	5.13×10^{-01}
Passive smoking exposure during pregnancy	Pseudomonas	Yes	-0.0497	1.12×10^{-01}	5.76×10^{-01}
Passive smoking exposure during pregnancy	Staphylococcus	Yes	0.0074	8.50×10^{-01}	9.41×10^{-01}
Passive smoking exposure during pregnancy	Streptococcus	Yes	0.0230	4.38×10^{-01}	7.37×10^{-01}

**Metadata**	**Phylum**	**Value**	**Coefficient**	**P-value**	**Q-value**
Conception group	Actinobacteria	Spontaneous	0.0140	1.36×10^{-01}	4.94×10^{-01}
Conception group	Bacteroidetes	Spontaneous	0.0830	6.21×10^{-06}	2.49×10^{-04}
Conception group	Cyanobacteria	Spontaneous	0.0156	6.79×10^{-02}	3.88×10^{-01}
Conception group	Firmicutes	Spontaneous	-0.0637	1.14×10^{-01}	4.66×10^{-01}
Conception group	Proteobacteria	Spontaneous	-0.0124	7.67×10^{-01}	9.38×10^{-01}
Maternal age (years)	Actinobacteria	/	0.0022	5.89×10^{-01}	8.91×10^{-01}
Maternal age (years)	Bacteroidetes	/	-0.0020	7.95×10^{-01}	9.38×10^{-01}
Maternal age (years)	Cyanobacteria	/	0.0057	1.16×10^{-01}	4.66×10^{-01}
Maternal age (years)	Firmicutes	/	0.0159	3.54×10^{-01}	7.70×10^{-01}
Maternal age (years)	Proteobacteria	/	-0.0225	2.11×10^{-01}	6.26×10^{-01}
Infant gender	Actinobacteria	Male	-0.0009	9.09×10^{-01}	9.38×10^{-01}
Infant gender	Bacteroidetes	Male	0.0193	1.93×10^{-01}	6.26×10^{-01}
Infant gender	Cyanobacteria	Male	0.0039	5.76×10^{-01}	8.91×10^{-01}
Infant gender	Firmicutes	Male	0.0260	4.34×10^{-01}	7.89×10^{-01}
Infant gender	Proteobacteria	Male	-0.0413	2.35×10^{-01}	6.26×10^{-01}
Gestational age (weeks)	Actinobacteria	/	-0.0030	4.81×10^{-01}	8.36×10^{-01}

Gestational age (weeks)	Bacteroidetes	/	-0.0041	6.15×10^{-01}	8.91×10^{-01}
Gestational age (weeks)	Cyanobacteria	/	0.0006	8.76×10^{-01}	9.38×10^{-01}
Gestational age (weeks)	Firmicutes	/	0.0222	2.28×10^{-01}	6.26×10^{-01}
Gestational age (weeks)	Proteobacteria	/	-0.0174	3.66×10^{-01}	7.70×10^{-01}
Gravidity	Actinobacteria	> 0	-0.0064	4.25×10^{-01}	7.89×10^{-01}
Gravidity	Bacteroidetes	> 0	-0.0036	8.13×10^{-01}	9.38×10^{-01}
Gravidity	Cyanobacteria	> 0	-0.0016	8.22×10^{-01}	9.38×10^{-01}
Gravidity	Firmicutes	> 0	-0.0114	7.40×10^{-01}	9.38×10^{-01}
Gravidity	Proteobacteria	> 0	0.0132	7.12×10^{-01}	9.38×10^{-01}
Mode of delivery	Actinobacteria	Vaginal	-0.0015	8.71×10^{-01}	9.38×10^{-01}
Mode of delivery	Bacteroidetes	Vaginal	-0.0710	1.15×10^{-04}	2.29×10^{-03}
Mode of delivery	Cyanobacteria	Vaginal	-0.0246	4.69×10^{-03}	4.69×10^{-02}
Mode of delivery	Firmicutes	Vaginal	-0.0687	9.12×10^{-02}	4.56×10^{-01}
Mode of delivery	Proteobacteria	Vaginal	0.1238	3.84×10^{-03}	4.69×10^{-02}
Maternal pre-pregnancy BMI (kg/m^{2})	Actinobacteria	/	0.0019	6.24×10^{-01}	8.91×10^{-01}
Maternal pre-pregnancy BMI (kg/m^{2})	Bacteroidetes	/	-0.0002	9.76×10^{-01}	9.76×10^{-01}
Maternal pre-pregnancy BMI (kg/m$^{2)}$	Cyanobacteria	/	0.0011	7.67×10^{-01}	9.38×10^{-01}
Maternal pre-pregnancy BMI (kg/m$^{2)}$	Firmicutes	/	-0.0190	2.61×10^{-01}	6.29×10^{-01}
Maternal pre-pregnancy BMI (kg/m$^{2)}$	Proteobacteria	/	0.0196	2.67×10^{-01}	6.29×10^{-01}
Passive smoking exposure during pregnancy	Actinobacteria	Yes	-0.0069	3.85×10^{-01}	7.70×10^{-01}
Passive smoking exposure during pregnancy	Bacteroidetes	Yes	-0.0300	4.92×10^{-02}	3.28×10^{-01}
Passive smoking exposure during pregnancy	Cyanobacteria	Yes	-0.0161	2.67×10^{-02}	2.14×10^{-01}
Passive smoking exposure during pregnancy	Firmicutes	Yes	0.0036	9.15×10^{-01}	9.38×10^{-01}
Passive smoking exposure during pregnancy	Proteobacteria	Yes	0.0236	5.06×10^{-01}	8.44×10^{-01}

Supplementary Table 2. Linear association analysis of infant first-pass meconium microbiota α-diversity with early-life factors

Factor	Diversity (Simpson index) in all meconium[a]		Diversity (Simpson index) in ART meconium[a]		Diversity (Simpson index) in Spontaneous meconium[a]	
	β (95% CI)	*P* value	β (95% CI)	*P* value	β (95% CI)	*P* value
Conception group						
ART	Ref.					
Spontaneous	0.12(0.03,0.21)	9.02×10^{-03}				
Maternal Age	-0.004(-0.01,0.01)	4.14×10^{-01}	-0.01(-0.03,0.0001)	5.43×10^{-02}	0.005(-0.01,0.02)	4.53×10^{-01}
Pre-pregnancy BMI (kg/m^2)	0.01(-0.01,0.02)	3.66×10^{-01}	-0.01(-0.03,0.01)	3.63×10^{-01}	0.01(-0.002,0.02)	9.36×10^{-02}
Gravidity						
0	Ref.		Ref.		Ref.	
> 0	-0.02(-0.10,0.06)	6.55×10^{-01}	-0.05(-0.15,0.06)	4.03×10^{-01}	0.004(-0.11,0.12)	9.42×10^{-01}
Gestational age (weeks)	0.02(-0.003,0.05)	8.30×10^{-02}	0.01(-0.03,0.04)	6.32×10^{-01}	0.02(-0.04,0.08)	4.61×10^{-01}
Mode of Delivery						
C/S	Ref.		Ref.		Ref.	
Vaginal	-0.08(-0.16,0.01)	7.43×10^{-02}	-0.04(-0.19,0.11)	6.29×10^{-01}	-0.17(-0.28,-0.06)	2.64×10^{-03}
Infant Gender						
Male	Ref.		Ref.		Ref.	
Female	0.02(-0.05,0.10)	5.52×10^{-01}	0.05(-0.05,0.16)	3.40×10^{-01}	-0.01(-0.12,0.09)	8.15×10^{-01}

[a]Adjusted for gestational age, mode of delivery and infant gender

Supplementary Table 3. Tobit regression analysis of infant first-pass meconium microbiota Bacteroidetes relative abundance with early-life factors

**Factor**	**Bacteroidetes abundance in all meconium[a]**		**Bacteroidetes abundance in ART meconium[a]**		**Bacteroidetes abundance in Spontaneous meconium[a]**	
	**β (95% CI)**	***P* value**	**β (95% CI)**	***P* value**	**β (95% CI)**	***P* value**
Conception group						
ART	Ref.					
Spontaneous	0.11(0.05,0.18)	4.02×10^{-04}				
Maternal Age	0.003(-0.004,0.01)	4.73×10^{-01}	0.002(-0.005,0.01)	5.36×10^{-01}	0.001(-0.01,0.01)	8.18×10^{-01}
Pre-pregnancy BMI (kg/m^2)	0.002(-0.01,0.01)	5.70×10^{-01}	0.001(-0.01,0.01)	8.39×10^{-01}	0.001(-0.01,0.01)	8.24×10^{-01}
Gravidity						
0	Ref.		Ref.		Ref.	
> 0	0.01(-0.05,0.06)	8.33×10^{-01}	-0.05(-0.11,-0.002)	4.11×10^{-02}	0.09(-0.01,0.19)	9.02×10^{-02}
Gestational age (weeks)	0.01(-0.01,0.03)	3.49×10^{-01}	-0.01(-0.02,0.01)	4.84×10^{-01}	0.01(-0.04,0.07)	6.85×10^{-01}
Mode of Delivery						
C/S	Ref.		Ref.		Ref.	
Vaginal	-0.07(-0.13,-0.01)	2.16×10^{-02}	-0.02(-0.09,0.06)	6.71×10^{-01}	-0.18(-0.28,-0.08)	3.99×10^{-04}
Infant Gender						
Male	Ref.		Ref.		Ref.	
Female	-0.02(-0.08,0.03)	3.86×10^{-01}	-0.002(-0.05,0.05)	9.45×10^{-01}	-0.04(-0.14,0.05)	3.76×10^{-01}

[a]Adjusted for gestational age, mode of delivery and infant gender
